# CD93 gene polymorphism is associated with disseminated colorectal cancer

**DOI:** 10.1007/s00384-015-2247-1

**Published:** 2015-05-26

**Authors:** Renate S. Olsen, Mikael Lindh, Emina Vorkapic, Roland E. Andersson, Niklas Zar, Sture Löfgren, Jan Dimberg, Andreas Matussek, Dick Wågsäter

**Affiliations:** Division of Drug Research, Department of Medical and Health Sciences, Faculty of Health Sciences, University of Linköping, 58185 Linköping, Sweden; Department of Laboratory Services/Division of Medical Diagnostics, County Hospital Ryhov, Building E3, Level 4, 44185 Jönköping, Sweden; Department of Surgery, County Hospital Ryhov, 44185 Jönköping, Sweden; Department of Natural Science and Biomedicine, School of Health Sciences, 55111 Jönköping, Sweden

**Keywords:** Biomarker, Colorectal cancer, Genotype, Prognosis, Single nucleotide polymorphism, Survival

## Abstract

**Purpose:**

Cluster of differentiation 93 (CD93) is involved in apoptosis and inflammation and has a suggested role in angiogenesis, and all of which are involved in the development and dissemination of cancer. We evaluated the expression of CD93 and the association with two single nucleotide polymorphisms (SNPs), rs2749812 and rs2749817, as possible biomarkers in colorectal cancer (CRC).

**Methods:**

Tissue levels and plasma levels of CD93 were measured using an enzyme-linked immunosorbent assay (ELISA). Expression of CD93 was determined by immunohistochemistry, western blot and gene expression analysis. Genotype frequencies were established for the SNPs by real-time polymerase chain reaction (PCR), and the association with tumour stage and survival was analysed.

**Results:**

Total CD93 levels were 82 % higher (*P* < 0.001) in tumours compared to matched normal tissues. Mean levels of soluble CD93 in plasma were 30 % lower (*P* < 0.001) in the patients compared to the controls. The T/T genotype of SNP rs2749817 was more common in stage IV patients, with consequently higher risk of CRC death (T/T vs. C/C and C/T; hazard ratio (HR) = 1.73, 95 % confidence interval (CI) = 1.11–2.67, *P* = 0.014), and was associated with a higher risk of CRC recurrence after radical operation (T/T vs. C/C and C/T; HR = 2.07, CI = 1.22–3.51, *P* = 0.007).

**Conclusions:**

We showed that the T/T genotype of SNP rs2749817 is associated with disseminated cancer at diagnosis and an increased recurrence rate after radical operation. Patients with this genotype may benefit from early identification.

## Introduction

Colorectal cancer (CRC) is the fourth most common cause of cancer mortality worldwide [1]. Local immunoregulation mediated by infiltration of inflammatory cells into colorectal adenocarcinomas and the development of chronic inflammation is considered of importance for tumour progression [2]. There is a need of predictive and prognostic factors in CRC. Risk factors in stages II and III have been of particular interest to tailor adjuvant treatment for the individual patient and to minimize the risk of toxicity [3, 4]. In CRC therapy, Kirsten RAS (KRAS) mutation status is a predictive marker for non-response to epidermal growth factor receptor (EGFR)-targeted drugs and is currently the only biomarker used for the management of CRC [5]. It has also been suggested that inherited genetic variations in immune-related genes, such as interleukin (IL)-4 and IL-6, may be associated with prognosis in CRC [6]. Although several biomarkers have been suggested for follow-up of treated CRC patients, their clinical application has not yet been fully evaluated [5, 7–9].

Human cluster of differentiation 93 (CD93) is an epidermal growth factor (EGF)-like domain containing transmembrane protein [10–12], which appears in two forms; a cell-associated full-length form and a truncated soluble form [13]. Expression of CD93 is seen on a variety of cell types involved in the inflammatory cascade and haematopoiesis [14, 15] and is predominantly expressed in the vascular endothelium [10]. Gene expression of CD93 has also been suggested to play a role in adhesion, phagocytosis and angiogenesis [10, 14–16]. Release and production of soluble CD93 also seems to be mediated by matrix metalloproteinases (MMPs) [17], but even proinflammatory cytokines such as tumor necrosis factor (TNF)-α has been proven to induce shedding of CD93 [10]. A correlation between a single nucleotide polymorphism (SNP) of CD93, rs2749812, and the risk of myocardial infarction (MI) and coronary heart disease (CHD) has been reported [18–20], and a reduced expression of soluble CD93 in plasma has been suggested as a potential biomarker for MI among Swedish patients with coronary artery disease (CAD) [20]. Recently, another study showed higher soluble levels of CD93 among Korean patients with acute MI, and they concluded that circulating CD93 might be a marker of monocyte inflammation in these patients [21]. These different results might be depended on the study design and ethnical differences. In an American study, high expression of CD93 was associated with higher survival rate among patients with multiple myeloma (MM) treated with bortezomib, indicating that high levels of CD93 may be a possible marker for better outcome in these patients [22]. Also, an association between SNP, rs2749817, of CD93 and the risk of breast cancer among Korean women has previously been described [23].

The aim of this study was to evaluate the expression of CD93 and the two SNPs of CD93 (rs2749812 and rs2749817) as possible candidate biomarkers of CRC.

## Patients and methods

### Study population

This study involved analysis of tissue sections, tissue lysates, plasma, ribonucleic acid (RNA) and deoxyribonucleic acid (DNA) samples from CRC patients from southeastern Sweden who had undergone surgical resections for primary colorectal adenocarcinomas at the Department of Surgery, County Hospital Ryhov, Jönköping, Sweden, during 1996 through 2012. The clinicopathological characteristics of the patients were obtained from surgical and pathological records. Follow-up was made through the medical records from all hospital departments and the primary care up to December 31, 2013. The date of an eventual recurrence and the date and cause of death as related to colorectal cancer or not were determined from a review of the patient’s files.

The patient group comprised 370 patients, including 197 males and 173 females with a mean age of 70 years (range 25–93). The control group comprised 316 blood donors, including 188 males and 128 females, with no known CRC history and from the same geographical region as the patients with a mean age of 61 years (range 41–70). The study was approved by the Local Ethics Committee of Linköping University, Sweden (Dnr. 2013/271-31), and informed consent was obtained from each patient.

### Tissue samples

Tumour and matched normal tissue samples were analysed from 101 of the 370 patients. The 101 patients consisted of 59 males and 42 females with a mean age of 70 years (range 36–90) with a tumour localized in the colon (*n* = 58) or rectum (*n* = 43). The tumours were classified according to the American Joint Committee on Cancer (AJCC) classification system; stage I (*n* = 17), stage II (*n* = 30), stage III (*n* = 34) and stage IV (*n* = 20). During surgery, tumour tissue and adjacent normal mucosa (~5 cm from the tumour) were excised from each patient and immediately frozen at −70 °C until analysis. Frozen normal and tumour tissue were thawed and homogenized in an ice-cold lysis buffer as previously described [24]. The protein content of the lysate supernatant fluid was determined using the Bradford protein assay (Bio-Rad Laboratories, Ltd., Hertfordshire, UK).

### Plasma samples

Venous blood samples were collected during surgery and centrifuged within 1 h to separate plasma and blood cells. Plasma was stored at −70 °C until analysis. Plasma was available from 110 patients (60 males and 50 females) with a mean age of 69 years (range 36–90) and from 106 controls (65 males and 41 females) with a mean age of 60 years (range 41–68). The patient’s tumours were localized in the colon (*n* = 68) or rectum (*n* = 42) and were classified as stage I (*n* = 17), stage II (*n* = 30), stage III (*n* = 42) and stage IV (*n* = 21).

### Western blot

Proteins in both tumour and matched normal tissue lysates were separated under reducing conditions by electrophoresis in a Novex 4–12 % Tris-Glycine Gel (Invitrogen/Life Technologies Ltd., Paisley, UK) using a standard protocol. In brief, separated proteins were transferred onto polyvinylidene difluoride (PVDF) transfer membranes (Amersham, Buckinghamshire, UK), blocked for 1 h with 5 % (wt/vol) non-fat dry milk and probed overnight with goat anti-human C1qR1/CD93 antibody (1 μg/mL; R&D Systems, Inc., MN, USA). After extensive washing, the blots were re-incubated for 1 h at room temperature with bovine horseradish peroxidase-conjugated anti-goat IgG secondary antibody (0.1 μg/mL; Santa Cruz Biotechnology, Santa Cruz, CA, USA). Blots were developed using an enhanced chemiluminescence Prime Western Blotting Detection Reagent system (Amersham).

### Immunohistochemistry

CD93 staining was performed using a standard protocol on 4 μm sections from formalin-fixed paraffin-embedded tissue blocks as previously described [24]. Sections were subsequently incubated with a primary goat anti-human C1qR1/CD93 antibody (0.2 μg/mL; R&D Systems) overnight at 4 °C and then with a horse secondary biotinylated affinity-purified anti-goat IgG antibody (1.5 μg/mL; Vector Laboratories, Ltd., Burlingame, CA, USA). Avidin-biotin peroxidase complexes (Vector laboratories) were added followed by visualization with 3.3′-diaminobenzidine (Vector Laboratories). Sections were counterstained with haematoxylin (Vector laboratories) and rehydrated before coverslips were added. Microscopy of the sections was performed using a Zeiss light microscope (Carl Zeiss Microscopy GmbH, Göttingen, Germany) along with the Zen lite software (Zeiss).

### Quantification of CD93 in tissue lysates and plasma

CD93 was measured in tissue lysates and plasma using a commercial enzyme-linked immunosorbent assay (ELISA) kit (R&D Systems Europe, Ltd., UK) and performed as previously described [20]. Tissue levels and plasma levels were determined using the Sunrise Tecan Microplate Reader (Tecan Austria GmbH, Salzburg, Austria) along with the Magellan 7.x 2010 software (Tecan). Tissue levels of CD93 in lysates were expressed as nanogram per milligram (ng/mg) of total CD93 and plasma levels of soluble CD93 as nanogram per millilitre (ng/mL).

### RNA extraction, cDNA synthesis and RNA quantification

Total RNA from 14 matched tumour and normal tissue biopsies was isolated using the RNeasy kit (Qiagen, Hilden, Germany). Complementary DNA (cDNA) amplification and real-time polymerase chain reaction (PCR) analysis were performed as previously described [25]. The CD93 (Life Technologies) expression results were normalized to the values of human glyceraldehyde-3-phosphate dehydrogenase (GAPDH), large ribosomal protein (RPLP0) and TATA-binding protein (TBP) (Life Technologies).

### DNA extraction and genotype determination

Whole blood DNA was extracted from 356 patients and from 305 controls for analysis of the SNPs rs2749817 and rs2749812. DNA was extracted using a QiaAmp DNA blood kit (Qiagen), and a TaqMan SNP genotyping assay was used for the analysis of rs2749812 and rs2749817 genotypes (Applied Biosystems/Life Technologies). DNA (10 ng) was mixed with TaqMan Universal PCR Master mix II (Applied Biosystems) and amplified using the 7500 Fast Real-Time PCR system (Applied Biosystems). All the patients were available with registered follow-up regarding survival, stadium (stage I = 64, stage II = 130, stage III = 108 and stage IV = 54) and localization (colon = 189 and rectum = 167).

### Statistical analysis

Differences in the frequencies of the CD93 SNPs between patients and controls and the association with localization and stage of the tumour were analysed by the *chi*-squared test. The Hardy-Weinberg equilibrium was tested for the genotypes. Differences in CD93 expression between tumour and matched normal tissues were examined by the Wilcoxon’s signed rank test, while differences in plasma CD93 levels between patients and controls were tested by the Mann-Whitney *U* test. These analyses were performed using the SPSS for Windows computer package (IBM SPSS Statistics, 2012, version 19, SPSS Inc., Chicago, IL, USA). Cancer-specific and disease-free survival analysis was performed by Cox’s regression and Kaplan-Meier analysis with the log-rank test, using the STATA 12 software (StataCorp, 2009, Stata Statistical Software: Release 12, College Station, TX, USA). The results were considered as statistically significant at *P* < 0.05.

## Results

### Localization and expression of CD93 in tumour and normal tissue

**Fig. 1:**
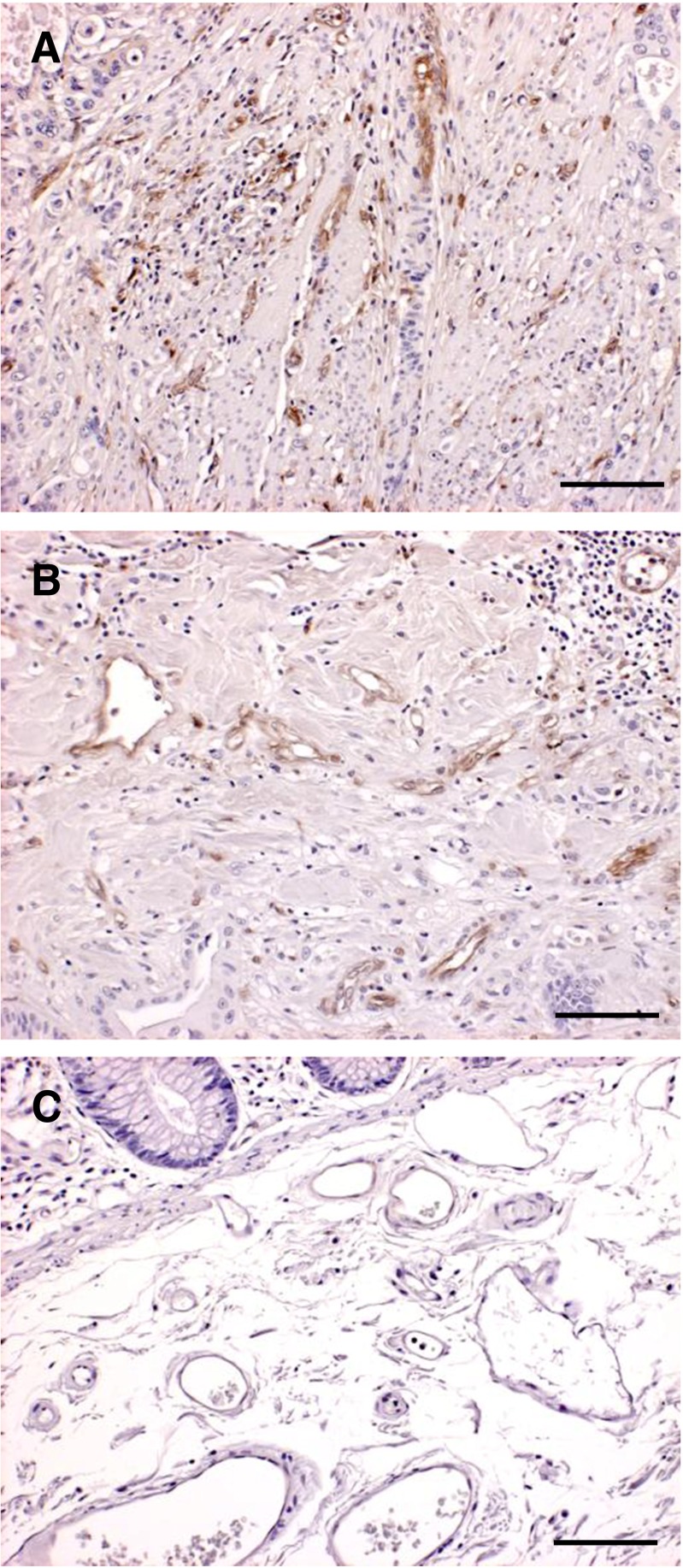
Immunohistochemical staining of CD93 in human CRC (**a**, **b**) and normal tissue (**c**). In tumour tissue, CD93 is present in endothelial cells located around small (**a**) and large blood vessels (**b**). In normal tissue, only faint CD93 expression in some vascular endothelial cells located around blood vessels is seen (**c**), *scale bar* = 20 μm

**Fig. 2:**
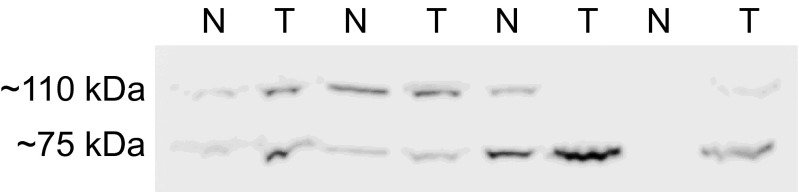
Western blot analysis of CD93 protein expression (~75 and ~110 kDa) in four representative specimens of human colorectal tumour and matched normal tissues; normal tissue (*N*) and tumour tissue (*T*)

**Fig. 3:**
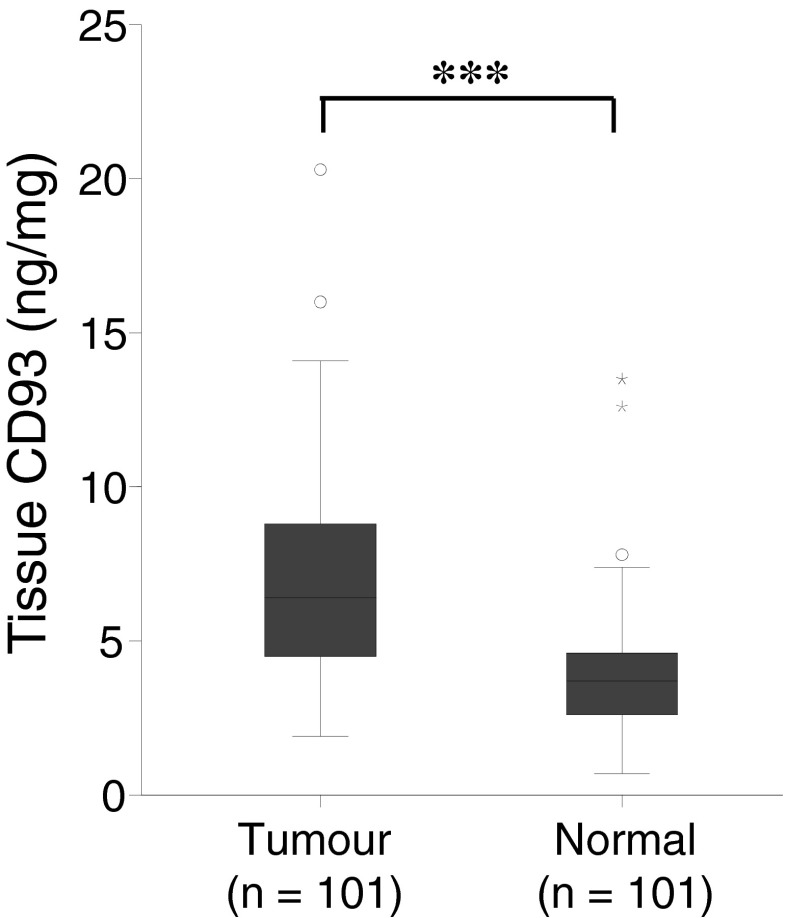
Tissue expression of total CD93 (ng/mg) from tumour and matched normal tissues in 101 CRC patients examined by ELISA; *asterisks* indicate *P* < 0.001

Expression of CD93 was predominantly seen in endothelial cells around blood vessels in cancerous tissue (Fig. 1a, b). CD93 was less expressed in normal tissue, and only weak staining was observed in some vascular endothelial cells (Fig. 1c). No difference in gene expression of CD93 was noted between matched normal or cancerous tissue (data not shown). Western blot analysis revealed a heterogeneous expression of CD93 in both normal and cancerous tissue, with two bands representing full length and truncated forms of CD93. The proteins had an approximate size of 75 and 110 kDa (Fig. 2). Quantification of total CD93 in the tissue lysates, using ELISA, showed a significantly 82 % higher expression in tumour tissues (mean, 7.1; standard deviation (SD), 3.33 ng/mg) compared to matched normal tissues (mean, 3.9; SD, 2.04 ng/mg) (*P* < 0.001) (Fig. 3). Eighty-nine out of the 101 patients had a significantly (*P* < 0.001) higher expression of CD93 in tumour tissue compared to matched normal tissue. The expression of total CD93 was not associated with stage, localization, gender or age (data not shown).

### Levels of CD93 in plasma

**Fig. 4:**
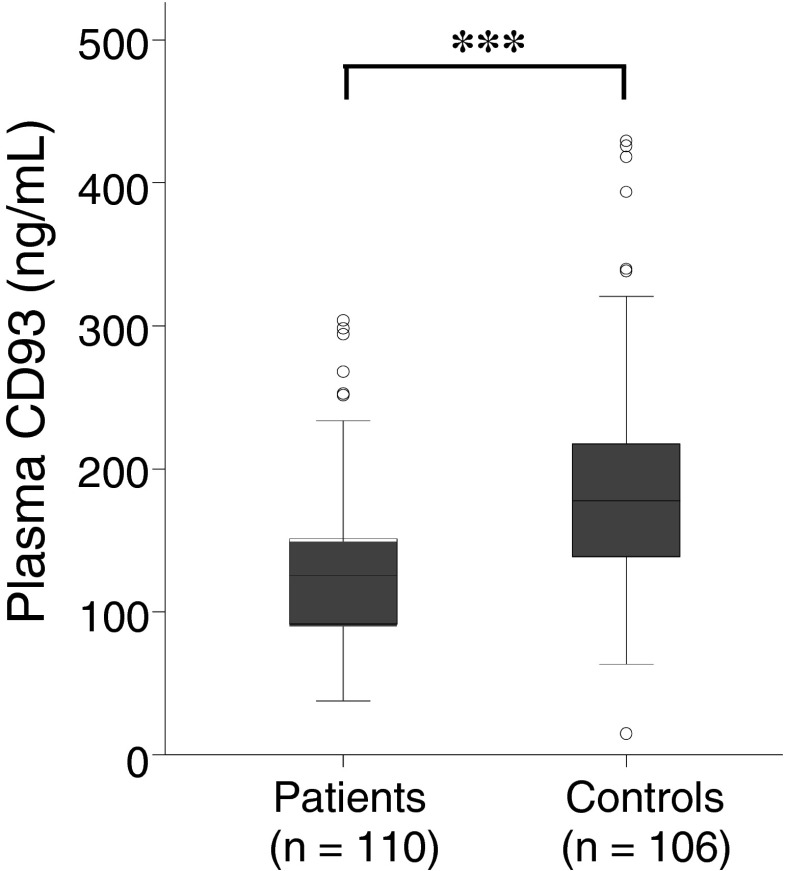
Plasma expression of soluble CD93 (ng/mL) from 110 CRC patients and 106 healthy controls examined by ELISA; *asterisks* indicate *P* < 0.001

The level of the soluble form of CD93 in plasma was significantly 30 % lower in the CRC patients (mean, 129.7; SD, 55.74 ng/mL) compared to the controls (mean, 186.0; SD, 74.47 ng/mL) (*P* < 0.001) (Fig. 4). No association was seen between level of soluble CD93 and stage, localization, gender or age (data not shown).

### Genotype frequencies and survival estimates

**Fig. 5:**
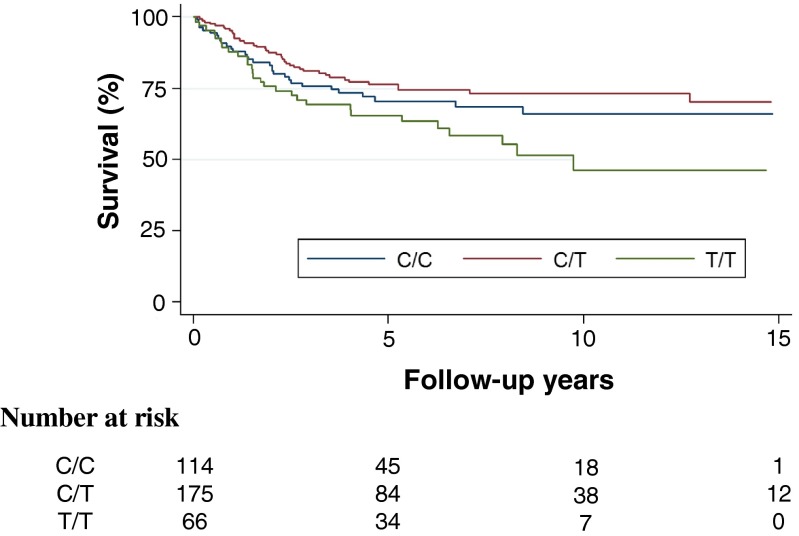
Kaplan-Meier curve describing cancer-specific survival estimates among CRC patients according to C/C (*middle blue line*), C/T (*upper red line*) and T/T (*lower green line*) genotypes of SNP rs2749817; *P* = 0.013

**Fig. 6:**
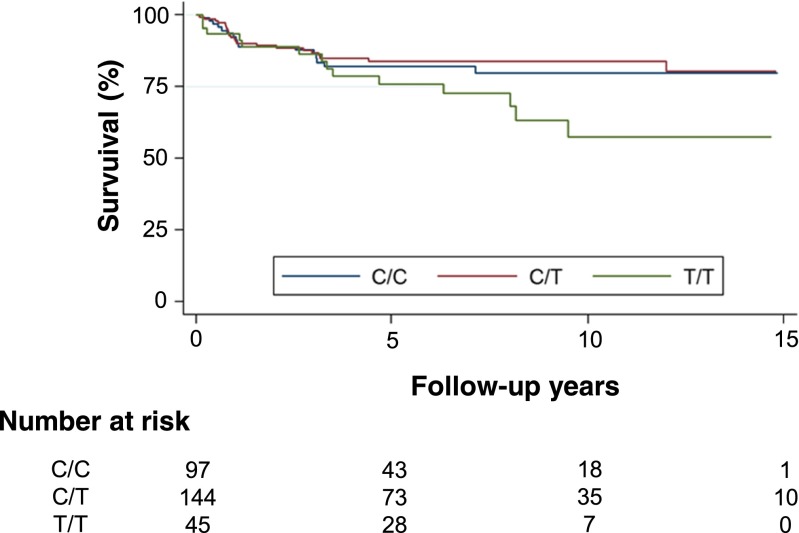
Kaplan-Meier curve describing disease-free survival estimates among CRC patients in stage I–III after R0 resection according to C/C (*middle blue line*), C/T (*upper red line*) and T/T (*lower green line*) genotypes of SNP rs2749817; *P* = 0.047

Genotype frequencies of SNP rs2749817 did not differ between the patients and controls (Table 1). Overall distribution of genotypes based on location and stage is described in Table 2. The distribution of the genotypes of SNP rs2749817 showed an increased frequency of the T/T genotype in stage IV (*P* = 0.009) when compared to stage I–III. No association between tumour site and the genotype distribution of SNP rs2749817 was seen (Table 2). The cancer specific survival differed between the genotypes of SNP rs2749817 (Fig. 5, log-rank test, *P* = 0.013). The T/T genotype had the highest risk of CRC death with a hazard ratio (HR) of 1.73 (95 % confidence interval (CI) = 1.11–2.67, *P* = 0.014) compared with the genotype C/C and C/T (Table 3). In patients that were tumour-free after operation (stage I–III and R0 resection), the T/T genotype had a worse prognosis, with a lower recurrence-free survival compared to patients with C/C and C/T genotype (Fig. 6, log-rank test, *P* = 0.047) and an increased risk of recurrence in CRC (Table 4, HR = 2.07, CI = 1.22–3.51, *P* = 0.007). When comparing the genotype frequencies of SNP rs2749817 to tissue levels of total CD93, the tumour tissue levels were significantly higher (20 %) among patients with T/T genotype (mean, 8.3; SD, 3.10 ng/mg) compared with C/C and C/T genotypes (mean, 6.9; SD, 3.36 ng/mg) (*P* = 0.037). There was no association in normal tissue levels (data not shown). We also examined the genotypes of SNP rs2749812, but no difference in genotype frequencies between patients and controls was observed. Among the patients, no association with stage and levels of CD93 was shown, and the genotypes of SNP rs2749812 did not have any effect on the survival of the CRC patients (data not shown).

**Table 1:** Genotype frequencies in numbers (%) of SNP rs2749817 in CRC patients and controls

Genotype	*Patients	*Controls
rs2749817	*n* = 356	*n* = 305
C/C	114 (32.0)	97 (31.8)
C/T	176 (49.4)	165 (54.1)
T/T	66 (18.6)	43 (14.1)

*Patients vs. controls is non-significant

**Table 2:** Genotype distribution of SNP rs2749817 in numbers (%) in 356 CRC patients in relation to tumour site and stage

Genotypes	Patients	C/C	C/T	T/T
*Tumour site				
Colon	189	62 (54.4)	93 (52.8)	34 (51.5)
Rectum	167	52 (45.6)	83 (47.2)	32 (48.5)
**Stage				
I	64	23 (35.9)	32 (50.0)	9 (14.1)
II	130	41 (31.5)	71 (54.6)	18 (13.9)
III	108	37 (34.3)	50 (46.3)	21 (19.4)
IV	54	13 (24.1)	23 (42.6)	18 (33.3)

*Tumour site is non-significant; **stage overall, *P* = 0.070; stages I–III vs. IV, *P* = 0.009

**Table 3:** Cancer specific mortality in CRC patients in stage I–IV

Genotypes	HR	95 % CI	*P* value
T/T vs. C/C	1.48	0.89–2.47	0.132
T/T vs. C/T	1.93	1.20–3.12	0.008
T/T vs. C/C and C/T	1.73	1.11–2.67	0.014

**Table 4:** Disease-free survival in CRC patients in stage I–III

Genotypes	HR	95 % CI	*P* value
T/T vs. C/C	2.02	1.06–3.85	0.034
T/T vs. C/T	2.12	1.19–3.76	0.010
T/T vs. C/C and C/T	2.07	1.22–3.51	0.007

Adjustment for localization, gender and age did not significantly affect these results for either of the SNPs.

## Discussion

New prognostic markers in CRC can help us to better understand the disease and to predict prognosis and can also serve as a basis for individualized therapy. In the present study, we found that (i) CD93 was increased and expressed in endothelial cells in blood vessels in CRC tissue; (ii) the plasma level of CD93 was lower in the CRC patients compared with the controls; (iii) the T/T genotype of rs2749817, a CD93-related SNP, was associated with disseminated cancer and an increased recurrence rate in patients that had the entire tumour removed at operation, which correlated to higher CD93 levels in the cancer.

During cancer development and progression, elevated inflammation is observed in cancer tissue due to the infiltration of leukocytes [26] and increased expression of proinflammatory cytokines. Also, chronic inflammation plays an important role as a risk factor in CRC [27]. CD93 is expressed on and shed from cells involved in the inflammatory cascade [28], and the release and production of soluble CD93 is enhanced during inflammation [29]. It has been speculated that the protein has a role in innate immunity and above all in apoptosis and inflammation [11, 30–33]. In our study, an increased expression of CD93 was observed in CRC tissue in the endothelial cells of blood vessels, while CD93 was found to a limited extent in blood vessels in normal tissue. Increased CD93 expression in endothelial cells could be involved in the response to local inflammation and on-going angiogenesis within the tumour. The involvement of CD93 in angiogenesis has previously been suggested in developing mice embryos [14], and the EGF-like domain of CD93 has been shown to stimulate angiogenesis in vitro in human umbilical vein endothelial cells (HUVEC) [10].

In our study, the levels of CD93 in plasma in the CRC patients were significantly lower compared to the controls. A low level of CD93 in plasma is surprising since CD93 is cleaved by MMPs and released, and MMPs are found in higher levels in CRC [17]. The low CD93 levels could indicate that only a small amount of CD93 is released from the tumour into the circulating blood of CRC patients due to unknown mechanisms or that the patients have an immunologic imbalance resulting in impaired production of CD93 from leucocyte and endothelial cells and/or restricts the secretion of CD93 from epithelial cells in colon and rectum. However, low CD93 levels in plasma have previously been found in patients with MI, a disease also involving an increased inflammatory response and MMPs [20]. The majority of the CRC patients (65.5 %) had soluble CD93 levels lower than 142 ng/mL, a threshold which was previously associated with increased risk for MI among a Swedish patient population with CAD [20]. Whether the low plasma levels of CD93 in patients is a significant level for both risk for cardiovascular diseases and CRC, or whether it reflects similar physiological and pathological mechanisms in the regulation of CD93 need to be further investigated.

The development of molecular markers has started to show promising results, but only KRAS mutation status has been accepted as a predictive marker for CRC and the response to drug treatment [5]. Different genes and SNPs have been correlated with clinical stage, presence of lymph node metastasis, clinical outcome, increased survival, follow-up after treatment and the risk of recurrence in CRC patients. These markers may be used as a supplement to the tumour lymph nodes metastasis (TNM) staging system, which alone cannot predict cancer prognosis [34–36].

We investigated the genotype distribution of two SNPs of the CD93 gene to see whether they were of any clinical importance in CRC. We showed that the T/T genotype of SNP rs2749817 is associated with disseminated cancer at diagnosis and increased risk of recurrence after radical operation, suggesting a lower resistance to the dissemination of tumour cells and reduced ability to control metastasis. No association between plasma CD93 levels and the genotypes of SNP rs2749817 in patients or controls was seen. However, this SNP has previously been studied among Korean women, and it was shown to have a correlation with breast cancer risk [23]. Hence, our results indicate that the T/T genotype of SNP rs2749817 may be used as a marker related to severe tumour disease and spread, even at the time of diagnosis, and may therefore be considered as a potential biomarker for outcome and survival in CRC. The exact mechanism of the T/T genotype is currently not known, but it is associated with increased tissue level of CD93 in the cancer that might affect angiogenesis, growth and spreading. The SNP rs2749817 is situated downstream of the 3′untranslated region (UTR) of the CD93 gene which could potentially affect translation and post translational modifications of the gene. The exact role of SNP rs2749817 on CRC survival remains unknown, but the data identifies the SNP as a risk marker for this variable.

Our data implies that identifying individuals with the T/T genotype at an early stage could be of importance both for outcome, follow-up and individualized treatment for CRC patients. Even though lower plasma levels of CD93 are found in both CRC and CAD patients, SNP rs2749817 is not associated with CAD, which indicates that this SNP will not be influenced by CAD [20].

The present study shows significantly higher CD93 levels in cancerous compared to matched normal tissues. Our observations also suggest that a low plasma concentration of CD93 may be a novel biomarker for CRC, but this must be further evaluated to be able to reveal the biological mechanism of plasma CD93 in CRC patients. Most importantly, a genotype belonging to an SNP of CD93 is correlated with disseminated CRC and may predict outcome for CRC patients.
